# Why do marionette lines appear? Exploring the anatomical perspectives and role of thread‐based interventions

**DOI:** 10.1111/srt.13676

**Published:** 2024-04-04

**Authors:** Gi‐Woong Hong, Soo‐Bin Kim, Soo Yeon Park, Jovian Wan, Kyu‐Ho Yi

**Affiliations:** ^1^ Samskin Plastic Surgery Clinic Seoul South Korea; ^2^ Division in Anatomy and Developmental Biology Department of Oral Biology Human Identification Research Institute BK21 FOUR Project Yonsei University College of Dentistry Seoul South Korea; ^3^ Made‐Young Plastic Surgery Clinic Seoul South Korea; ^4^ Asia Pacific Aesthetic Academy Hong Kong Hong Kong; ^5^ Maylin Clinic (Apgujeong) Seoul South Korea

**Keywords:** aging factors, facial anatomy, marionette lines, threads, treatment strategies

## Abstract

The pathogenesis of marionette lines involves a complex interplay of anatomical, physiological, and age‐related factors leading to the development of wrinkles around the oral commissures. This exploration delves into the distinct anatomical predispositions observed among different ethnicities, emphasizing the role of compact modiolus structures and muscle compositions. Notably, individuals of East Asian descent exhibit inherent facial structures that predispose them to pronounced sagging around the oral commissures during aging. The emergence of distinct facial lines, such as the commissural line and the melolabial fold, contributes to the formation of marionette lines. This specific wrinkle pattern, resembling a marionette puppet's mouth contours, is influenced by various factors like bone resorption, gravitational forces, fat compartment variations, muscle compression, ligament tethering, and skin aging. Treatment strategies for marionette lines encompass diverse interventions, including filler injections, botulinum neurotoxin, surgeries targeting fat reduction, thread lifting, and volumizing fillers. These approaches aim to address the underlying causes and mitigate the appearance of marionette lines. Botulinum neurotoxin injections, for instance, weaken specific facial muscles, reducing downward strain and aiding in tissue retraction. Anatomical considerations during procedures are crucial to avoid nerve or vascular damage. Delicate manipulation and precise entry points are essential to prevent inadvertent injuries, particularly concerning blood vessels like the facial artery and nerves like the mental nerve. Technical guidelines for procedures targeting marionette lines involve specific techniques like cogged thread reverse methods and volumizing thread placements. Attention to entry points, tissue engagement, and the direction of threads is crucial for effective treatment outcomes, minimizing complications, and ensuring patient safety.

## 1 INTRODUCTION

Facial aging is a complex process involving significant alterations in various facial structures. Skin changes, such as reduced collagen and loss of subcutaneous fat, along with transformations in the facial skeleton and soft tissues, contribute to this progression.^1^, ^2^, ^3^, ^4^ Presently, there is a wide array of facial rejuvenation methods available to address these aging effects. These sought‐after procedures, including fillers, toxins, thread lifting, lipofilling, and laser treatments, are highly in demand.^5^, ^6^ Recent research highlights a shift toward minimally invasive approaches in rejuvenation surgeries, preferred by both patients and surgeons due to their lower complication rates and shorter recovery periods.^7^, ^8^, ^9^, ^10^

Among these minimally invasive procedures, thread lifting stands out as a popular treatment modality for facial rejuvenation. Initially devised as a facelift technique, practitioners now widely consider thread lifting as a creative and practical method for skin rejuvenation or lifting.^11^, ^12^, ^13^, ^14^ Recent studies emphasize the reliability of thread lifting as a minimally invasive approach, showcasing early recovery, high patient satisfaction, and minimal risk of adverse effects.^13^, ^14^, ^15^, ^16^, ^17^, ^18^, ^19^, ^20^

Facial volume loss during the aging process significantly impacts the definition of the jawline, leading to skin sagging, jowls, and the development of prominent marionette lines. The jawline plays a crucial role in facial attractiveness, particularly in adults, necessitating a comprehensive approach to facial rejuvenation that includes evaluating and addressing jawline concerns in both men and women.^25^

Particularly, addressing the aging effects like marionette lines on facial skin is a common objective in cosmetic procedures. These lines, also known as melomental folds, result from facial aging and manifest as downward curving wrinkles originating from the corners of the mouth. Previously, the origin of marionette lines was attributed to repetitive facial movements and the impact of gravity on less elastic skin tissue. Treating well‐established marionette lines cosmetically has proven challenging. However, there's a noticeable shift away from using more invasive facelift methods to address these lines, favoring less invasive procedures that appear to be more effective. The use of threads has shown promise in enhancing the appearance of marionette lines.

## 2 THE PATHOGENESIS OF MARIONETTE LINES

**FIGURE 1:**
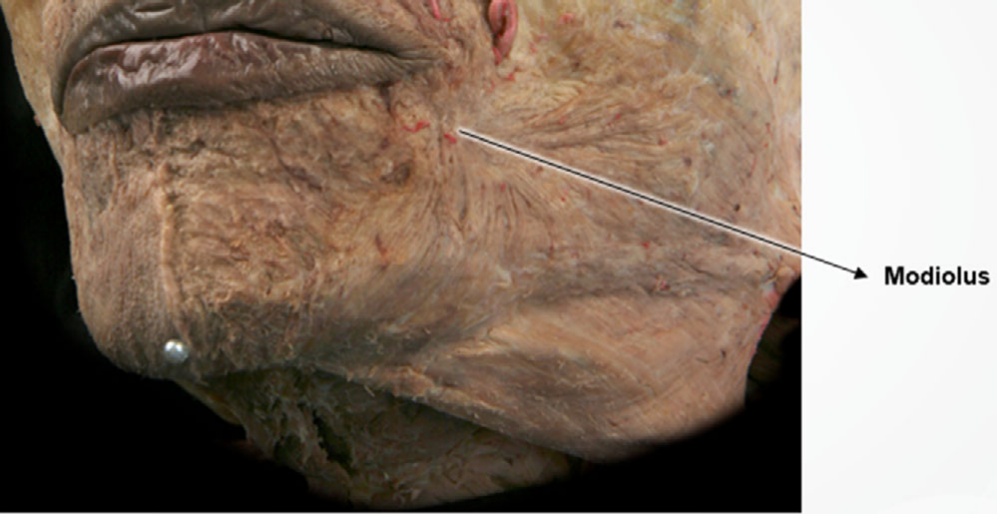
Spatial localization of the modiolus among individuals of Asian descent.

**FIGURE 2:**
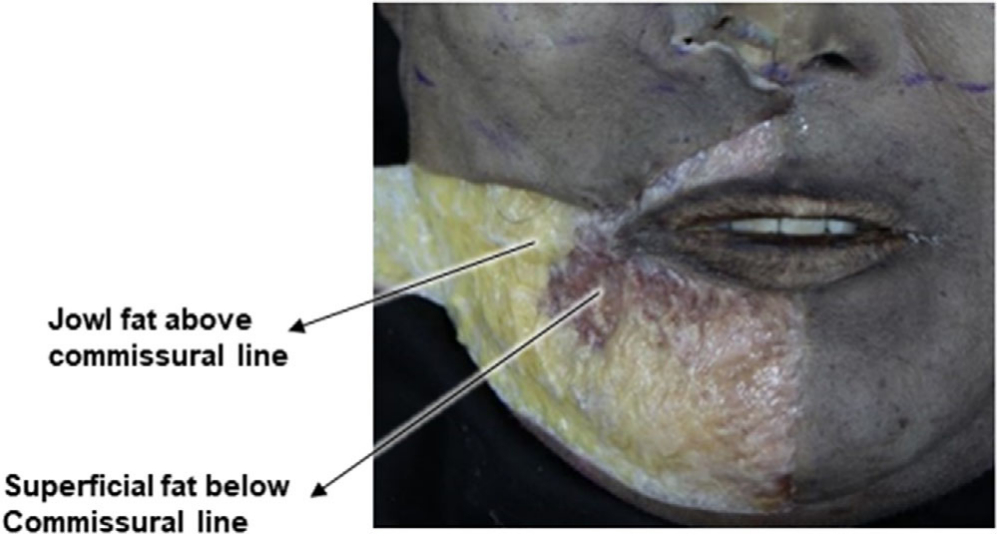
Disparities in dermal thickness encircling the perioral region.

An exploration of the etiological factors contributing to the development of wrinkles around the oral commissures is essential. In individuals of Caucasian or African descent, the presence of compact modiolus structures, small muscular formations formed by the convergence of facial muscles, is notable, primarily situated at or above the level of the oral commissures. Conversely, in East Asian populations, including Koreans, the modiolus typically positions itself approximately 11 mm laterally and about 9 mm inferiorly from the corners of the mouth. Consequently, individuals from these demographics commonly exhibit a natural predisposition where the oral commissures tend to visually descend, even during a neutral facial state—a characteristic more pronounced than observed in individuals of Western descent. This inherent anatomical disposition often results in increased susceptibility to visible sagging around the oral commissures during the aging process (Figure 1). Furthermore, among the muscles composing the modiolus, the depressor anguli oris muscle, situated most superficially, along with the superficial and deep layers of the orbicularis oris muscle positioned at intermediate depths, contribute to stratification due to the difference in their depth. A notable feature in East Asians is the emergence of the commissural line, a vertically slanted line that becomes pronounced when the corners of the mouth deepen, owing to the anatomical position of the modiolus. In instances where there is a deficiency in the lateral lower lip fat compartment, constituting the superficial fat beneath the lips, a discernible difference in thickness arises between the jowl fat, a superficial fat located toward the cheeks, and the tissues of the lower lip area. Consequently, due to this variation in thickness between the upper and lower tissue layers, the resultant commissural line becomes more prominent, leading to an occurrence of mouth corner drooping (Figure 2).

**FIGURE 3:**
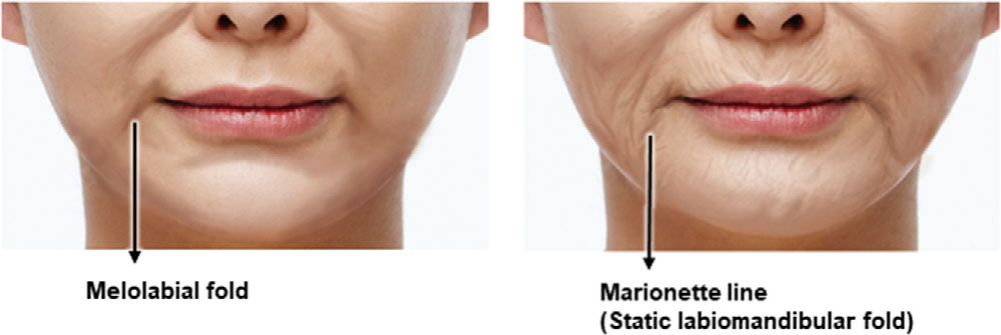
Depiction of the melolabial fold and marionette line.

The delineation of the superficial anatomical boundary between the cheek and the lower lip region, known as the cheek‐chin crease or melolabial fold, becomes more pronounced as the discernible difference in tissue thickness between these two regions intensifies. This accentuation leads to a more prominent appearance of a downturned fold around the mouth, conveying a melancholic or somber expression (Figure 3).

**FIGURE 4:**
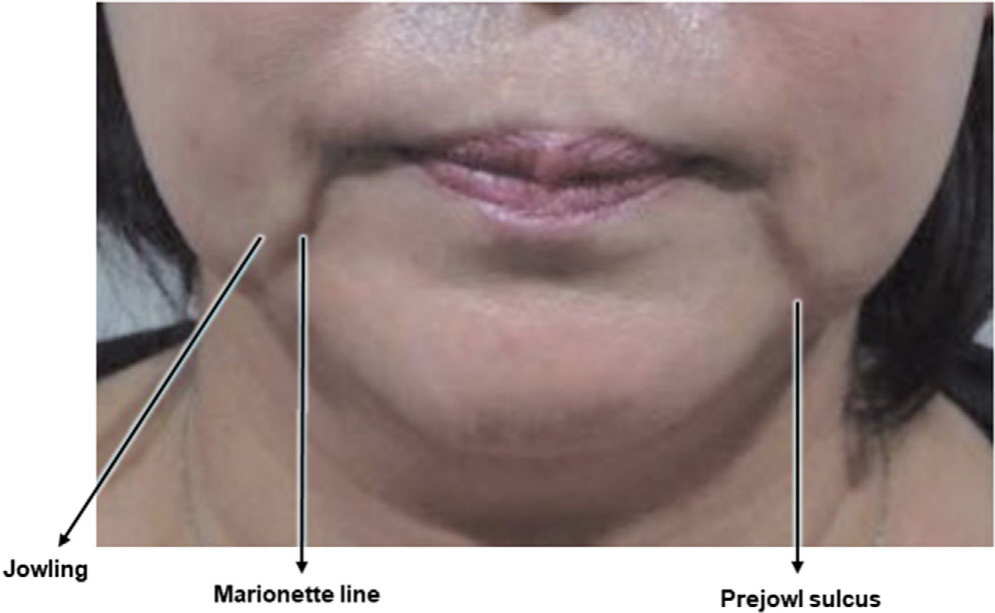
Predominant clinical manifestations characterizing the marionette line.

As individuals age, the melolabial fold deepens due to various factors, extending the wrinkle line toward the border of the mandible. The extended fold, situated in front of the jawline, tends to diminish unlike the protruding tissues above it, resulting in the formation of the prejowl sulcus. Commonly referred to as the “marionette line,” this particular wrinkle pattern resembles the mouth contours of a marionette puppet, popular in European puppet theater. The term “static labiomandibular fold” is also used due to its nature as a wrinkle extending obliquely from the area around the mouth to the jawline even in an expressionless face (Figure 4).

The delineation of the superficial anatomical boundary between the cheek and the lower lip region, known as the cheek‐chin crease or melolabial fold, becomes more pronounced as the discernible difference in tissue thickness between these two regions intensifies. This accentuation leads to a more prominent appearance of a downturned fold around the mouth, conveying a melancholic or somber expression (Figure 3).

With advancing age, the melolabial fold deepens due to various factors, extending the wrinkle line toward the border of the mandible. The extended fold, situated in front of the jawline, tends to diminish unlike the protruding tissues above it, resulting in the formation of the prejowl sulcus. Commonly referred to as the “marionette line,” this particular wrinkle pattern resembles the mouth contours of a marionette puppet, popular in European puppet theater. The term “static labiomandibular fold” is also used due to its nature as a wrinkle extending obliquely from the area around the mouth to the jawline even in an expressionless face (Figure 4). Factors contributing to the development of the marionette line include maxilla and mandible bone resorption, gravitational forces causing downward displacement, reduction of the deep fat layer beneath the depressor anguli oris muscle, compression of the depressor anguli oris muscle itself, the tethering effect of the mandibular ligament, sagging of redundant skin and connective tissue, and drooping of the jowl and buccal fat. Depending on the underlying causes, a combination of treatment strategies may be necessary.

**FIGURE 5:**
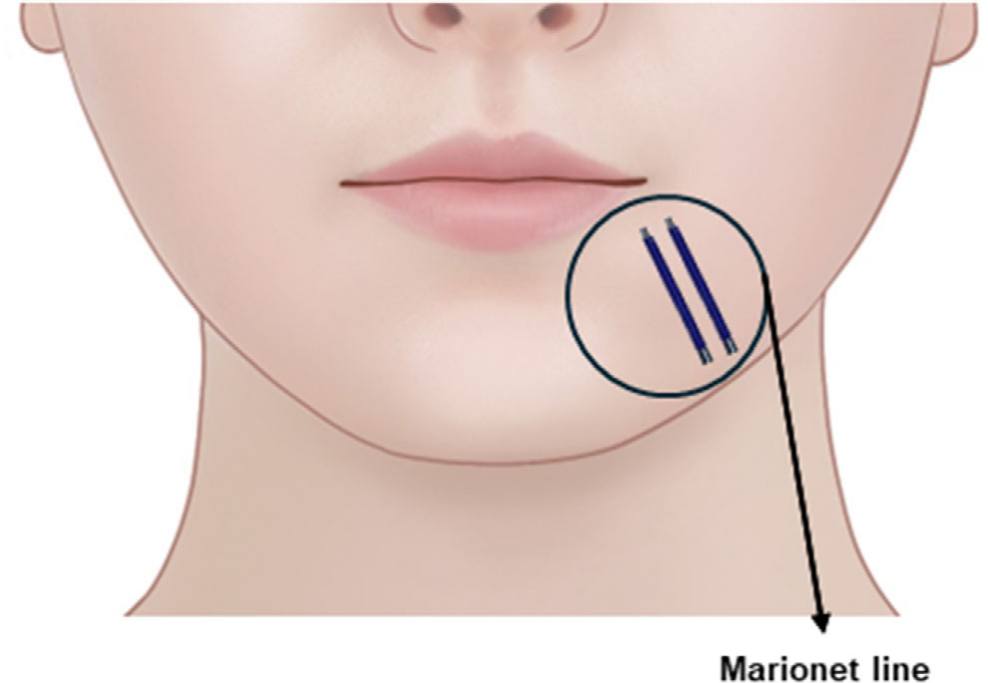
Intervention involving the augmentation of the sub‐marionette line area. Two volumizing thread (Licellvi Jamber F (JWorld Co., Ltd.) or countourel Jamber F (Croma Pharma GmbH) has been inserted below the marionette line for correction.

Interventions for addressing the marionette line extend beyond filler injections to address the sunken area in front of the marionette line and the unevenness of the mandible border line, including the prejowl sulcus. These interventions may involve botulinum neurotoxin injections to improve mouth corner depression caused by the contraction of the depressor anguli oris muscle. Additionally, procedures or surgeries aiming to reduce the volume of the protruding jowl and buccal fat, or techniques like thread lifting to tighten and smoothen the perioral area by pulling the sagging skin, fat, and surrounding tissues, are viable options. Furthermore, volumizing fillers can help lighten the lines formed on the skin around the mouth corner while simultaneously firming the skin to minimize folding during facial expressions (Figure 5). Among various procedures, the administration of botulinum neurotoxin, which weakens the depressor anguli oris muscle, contributes to reducing the force acting downward at the mouth corner. This action diminishes the strain exerted below the mouth corner, potentially aiding thread lifting to retract the lax tissues above the mouth corner crease. Moreover, the current trend in botulinum neurotoxin procedures encompasses techniques aimed at maintaining facial top‐bottom balance by slightly mitigating the downward‐pulling action of muscles, resulting in a relatively pleasing expression akin to a subtle smile.

## 3 ANATOMICAL CONSIDERATIONS

During the thread lifting procedure targeting the marionette line and jowl, attention should be given to the vasculature and nerves around the corners of the mouth, which are encountered by needles or cannulas. In this specific region, the facial artery emits branches that ascend toward the nasolabial fold, extending both above and below the corners of the mouth, alongside the jawline. Additionally, a premasseteric branch originates from the facial artery near the jawline, following along the anterior border of the masseter muscle. To prevent damage to these blood vessels, it is essential to employ delicate manipulation of the cannulas or needles during the procedure.

Moreover, when utilizing the cross technique with volume threads to address superficially located creases in the Marionette line area, careful selection of the entry point on the inner side of the Marionette line becomes imperative.^24^ This precaution is necessary to avoid inadvertent injury to the mental artery and nerve emanating from the mental foramen, situated beneath the corners of the mouth. The mental foramen often lies approximately midway along a vertical line drawn from the corners of the mouth to the borderline of the mandible.

## 4 TECHNICAL GUIDELINE

**FIGURE 6:**
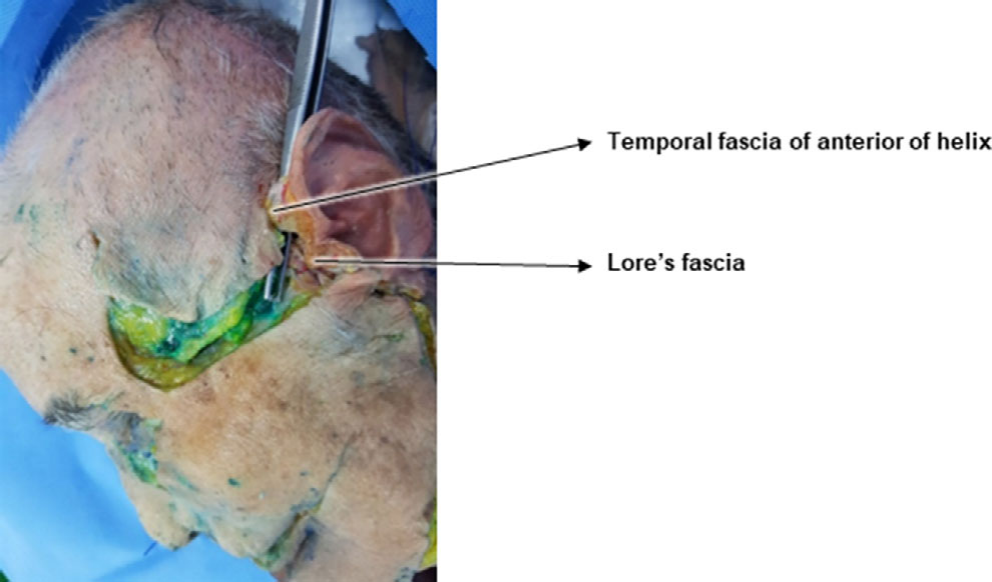
Identification and establishment of anchoring sites for the reverse technique employing I‐type bidirectional cogged threads.

The cogged thread reverse technique, employed to address marionette lines inclined toward the jawline and the protruding jowl fat, utilizes a vector oriented toward the ear, perpendicular to the direction of the fold. The entry point for the cogged thread insertion is positioned near the inferior margin of the jowl fat, vertically aligned above the marionette line. Optimal fixing points are near the temporal fascia close to the antihelix of the ear or around Lore's fascia in front of the tragus, approximately midway along the ear (Figure 6).

Following a needle puncture at the entry point, the practitioner elevates the jowl fat using the left hand before introducing the cannula. The cannula is then directed along the predetermined vector of the thread, traversing through the lower part of the elevated jowl fat, replicating the methodology employed in the reverse marionette line procedure.

In the lateral face, the practitioner follows the thick and firm superficial musculoaponeurotic system (SMAS) layer. Transitioning from the anterior to the lateral face, particularly beyond the vertical line of the lateral orbital rim, the denser SMAS may impart a more rigid sensation as the cannula traverses through.^21^, ^22^ When introducing the cannula into the entry point and engaging the target tissue, the objective is to effectively involve the jowl fat in its entirety. Should the cannula be inserted excessively deep and directed towards the buccal fat pad, there is a risk of inadvertent proximity to the oral mucosa. It is imperative to prevent the cannula's tip from pointing excessively downward. The recommended approach involves gently holding the elevated jowl fat and carefully guiding the cannula's tip into this fat, creating a sensation of envelopment.

**FIGURE 7:**
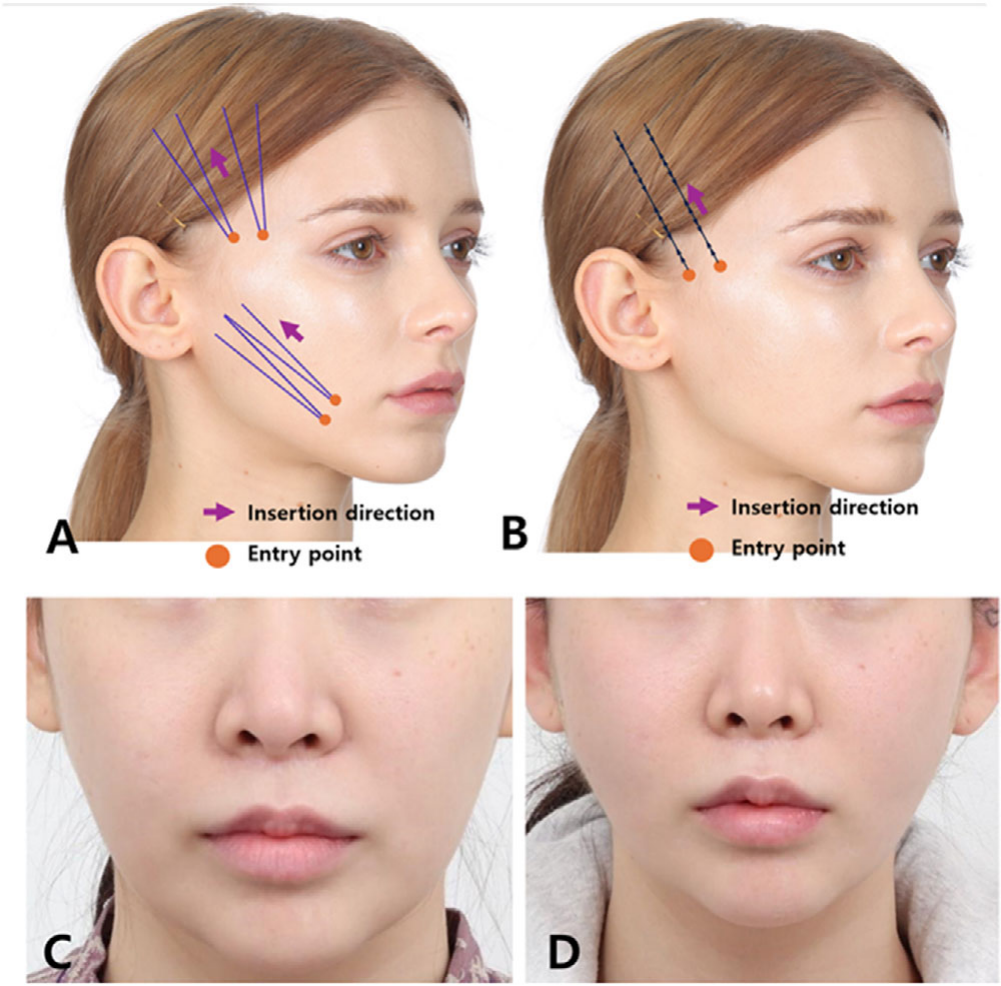
The techinique for correcting marionette line can be approached using various methods such as the top‐to‐bottom approach, the reverse technique, and the use of fixation threads. Specific guidance for the entry point and path for the reverse method employs I‐type cogged threads to address marionette lines. The upper temple can be primarily treated using the reverse technique with Bluerose Forte L (Hugel, Inc.), Licellvi X (JWorld Co., Ltd.), or countourel ultra (Croma Pharma GmbH) followed by a secondary application to elevate the marionette line (A). The fixation procedure is performed using Bluerose Multi (Hugel, Inc.), Licellvi Max (JWorld Co., Ltd.), or countourel max (Croma Pharma GmbH). A 32‐year‐old woman presented with marionette line concerns before (C) and after (D) undergoing thread lifting treatment.

When manipulating the jowl fat with fingers, it is imperative to elevate both the fat and the skin in front of the marionette line adequately. The region below the mouth corner is minimally affected by oral movements; therefore, maintaining the correct direction and depth of the thread's path, even with a slightly interior entry point relative to the marionette line, should not pose significant issues post‐procedure (Figure 7).

While the patient is in a reclined position with a slightly elevated chin, the practitioner observes a lifting of tissues above the marionette line toward the ear. During this posture, the specialized reverse‐cogged thread is inserted through the designated entry point, with the thread's thickness determined based on wrinkle severity, skin thickness, and the extent of movement around the mouth.

Within the region traversed by the barbed threads, vertically positioned fibrous tissues, identified as masseteric‐cutaneous ligaments, are present. These ligaments, along with the superficial muscular aponeurotic system (SMAS) on the outer aspect of the face, serve as anchoring points for the barbed threads. Consequently, the engagement of these fibrous tissues enhances tissue mobilization effects and prolongs the retention duration induced by the threads.

To mitigate the folded line between firm and lax tissues after uplifting the overall shape of the mouth corner by displacing lax tissues outside the marionette line upwards, the utilization of volumizing threads becomes an option. Employing a cross technique with volumizing threads entails creating a medial entry point relative to the wrinkle line. Inserting the agents perpendicular to the wrinkle line from below to above allows the volumizing threads within the thick tissue beneath the wrinkle line to act as a supporting base. This facilitates the upper part of the volumizing threads to exert pressure on and elevate the lax skin tissue above the wrinkle line.

**FIGURE 8:**
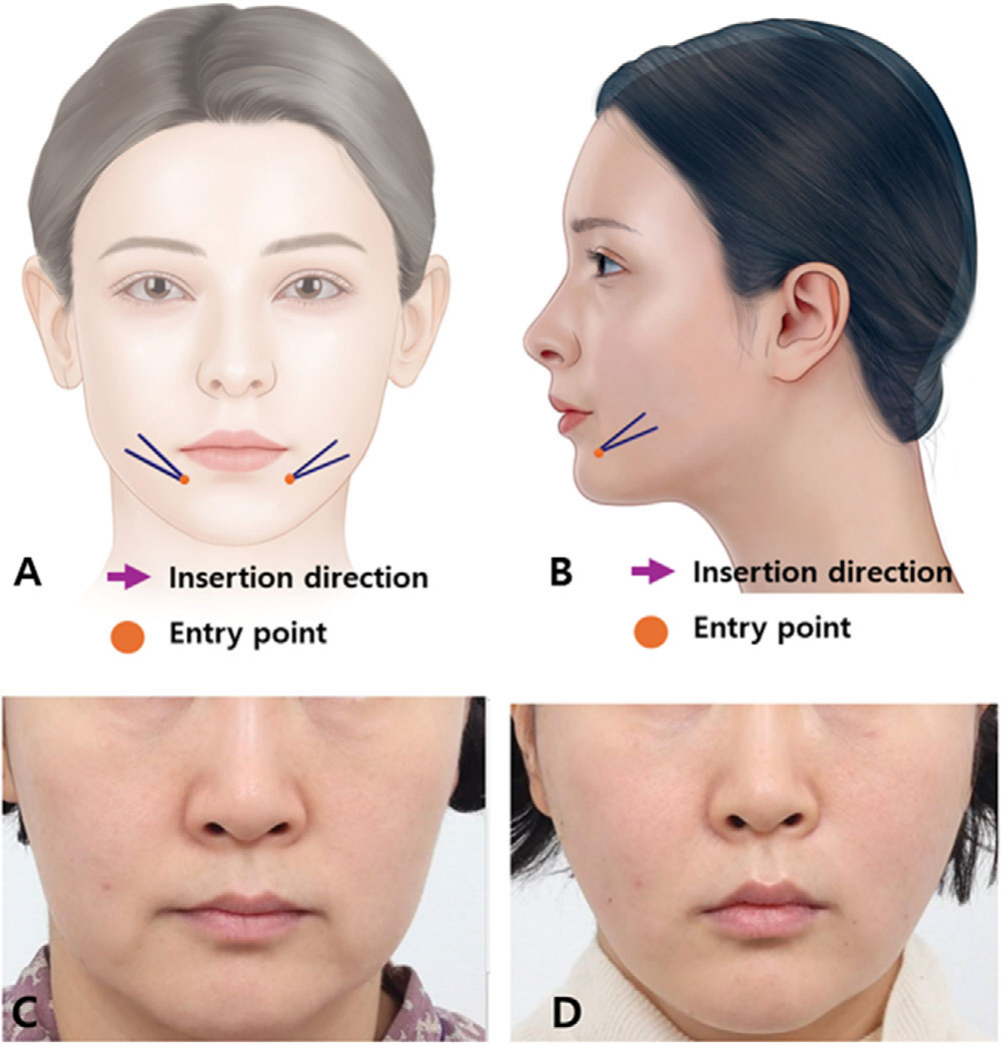
Guidance on insertion site and trajectory for the cross technique employing volumizing polydioxanone threads in the treatment of marionette lines. The thread used are Licellvi Jamber F (JWorld Co., Ltd) or countourel Jamber F (Croma Pharma GmbH). Frontal view (A) and lateral view (B). A 47‐year old women presented with marionette line concerns before (C) and after (D) undergoing thread lifting treatment.

By strategically placing several volumizing threads in the vertical direction of the wrinkle line, as illustrated, collagen production aligns with the agents, leading to increased firmness in the lax skin tissues above the wrinkled area (Figure 8). This process mitigates the degree to which the skin around the mouth corner is compressed and folded by facial muscles during lip movements, thereby minimizing the appearance of folds.

## 5 DISCUSSION

In today's aesthetic clinics, knotless thread lifting devices using absorbable threads have gained popularity. However, a significant limitation of this procedure is its suitability for only a moderate degree of facial tissue laxity.

The formation of marionette lines, influenced by facial anatomy and aging, results in visible sagging around the mouth. These lines, associated with various factors like muscle composition and tissue depth differences, contribute to a down‐turned appearance, resembling a marionette puppet's mouth. Addressing these lines often requires a comprehensive approach involving minimally invasive procedures, such as thread lifting and fillers, to minimize sagging and enhance facial appearance. Techniques focusing on specific anatomical considerations and careful insertion of threads have shown promise in effectively treating and minimizing these lines with reduced risk and improved outcomes.

Favorable anatomical traits for absorbable thread lifting include a low body mass index, minimal soft tissue fullness, sturdy underlying bony structures for support, and good skin quality. Conversely, individuals with obesity and thicker soft tissues tend to yield less favorable results, necessitating careful patient selection for optimal outcomes.

To address severe skin sagging, the insertion of 1 to 3 additional cog threads along the cheek proves effective. Meanwhile, for addressing additional marionette lines and lifting the chin, using 3 to 5 extra cog threads perpendicular to the chin line yields more favorable outcomes.

The PDO thread technique offers advantages such as avoiding general anesthesia and scarring due to its incision‐free nature. It has shown efficacy in treating uneven facial textures, midface slackness, and mild to moderate jowls in specific patient groups. Complications associated with this procedure are infrequent and generally minor.^20^, ^25^ Utilizing PDO threads for aesthetic facial rejuvenation and lifting is considered a safe method.

## CONFLICT OF INTEREST STATEMENT

I acknowledge that I have considered the conflict‐of‐interest statement included in the “Author Guidelines.” I hereby certify that, to the best of my knowledge, that no aspect of my current personal or professional situation might reasonably be expected to significantly affect my views on the subject I am presenting.

## Data Availability

The data that support the findings of this study are available from the corresponding author upon reasonable request.
